# Maintenance Deep Transcranial Magnetic Stimulation Sessions are Associated with Reduced Depressive Relapses in Patients with Unipolar or Bipolar Depression

**DOI:** 10.3389/fneur.2015.00016

**Published:** 2015-02-09

**Authors:** Chiara Rapinesi, Francesco Saverio Bersani, Georgios D. Kotzalidis, Claudio Imperatori, Antonio Del Casale, Simone Di Pietro, Vittoria R. Ferri, Daniele Serata, Ruggero N. Raccah, Abraham Zangen, Gloria Angeletti, Paolo Girardi

**Affiliations:** ^1^Department of Neurosciences, Mental Health, and Sensory Organs NESMOS, School of Medicine and Psychology, Sant’Andrea Hospital, Sapienza University of Rome, Rome, Italy; ^2^Neuropsychiatric Unit, Villa Rosa, Suore Ospedaliere of the Sacred Heart of Jesus, Viterbo, Italy; ^3^Department of Neurology and Psychiatry, Policlinico Umberto I University Hospital, Sapienza University of Rome, Rome, Italy; ^4^Department of Human Sciences, European University of Rome, Rome, Italy; ^5^Department of Psychiatric Rehabilitation, P. Alberto Mileno Onlus Foundation, Vasto, Italy; ^6^ATID Ltd – Advanced Technology Innovation Distribution, Rome, Italy; ^7^Department of Life Sciences, Ben Gurion University of the Negev, Be’er Sheva, Israel

**Keywords:** deep transcranial magnetic stimulation, bipolar disorder, neuropsychiatry, brain modulation, recurrent major depressive disorder

## Abstract

**Introduction:** Deep transcranial magnetic stimulation (dTMS) is a new form of TMS allowing safe stimulation of deep brain regions. The objective of this preliminary study was to assess the role of dTMS maintenance sessions in protecting patients with bipolar disorder (BD) or recurrent major depressive disorder (MDD) from developing depressive or manic relapses in a 12-month follow-up period.

**Methods:** Twenty-four drug-resistant patients with a current depressive episode and a diagnosis of MDD or BD have been enrolled in the study. All the participants underwent daily dTMS sessions for 4 weeks. One group (maintenance – M group) received additional maintenance dTMS sessions weekly or twice a week.

**Results:** After the first dTMS cycle, a significant reduction of Hamilton Depression Rating Scale (HDRS) scores was observed in all participants. Subsequently, the HDRS mean scores did not significantly change over time in the M group, while it significantly increased in the non-M-group after 6 and 12 months.

**Discussion:** This study confirms previous evidence of a positive therapeutic effect of dTMS on depressive symptoms and suggests that, after recovery from acute episodes, maintenance dTMS sessions may be helpful in maintaining euthymia in a 12-month follow-up period.

## Introduction

Repetitive transcranial magnetic stimulation (rTMS) is a non-invasive technique used to treat drug-resistant depressive episodes in major depressive disorder (MDD) (1) and bipolar disorder (BD) (2), as well as a range of other neurological and psychiatric conditions (3). rTMS therapy is administered through an electromagnetic coil placed above patient’s scalp; the coil sends magnetic pulses to the brain and induces a modulation of the electrical activity in the underlying cortex (3).

Neuroimaging studies have led to the imbalance hypothesis of depression, which postulates prefrontal asymmetry with relative hypoactivity in the left dorsolateral prefrontal cortex (DLPFC), associated with a relative hyperactivity in the right DLPFC (4). Consistently, patients with depression have been found to benefit from excitatory high-frequency (more than 1 Hz) rTMS over the left DLPFC and inhibitory low-frequency (<1 Hz) rTMS over the right DLPFC (5).

There is evidence that clinical depression involves dysfunction in integrated neural pathways linking cortical, subcortical, and limbic sites and related cellular and molecular mediators (6); it is supported that effective stimulation of this pathway would benefit patients with depressive symptoms, but also that the regions involved cannot be effectively stimulated utilizing standard rTMS technology (7). For this reason, a new coil (H-coil) has been developed to allow safer stimulation of deeper brain regions [Deep Transcranial Magnetic Stimulation (dTMS)] (8).

Several studies assessed the efficacy and safety of dTMS in MDD [for review, see Ref. (9)], and one study assessed it in BD depression (10). Studies suggested a positive therapeutic effect of dTMS in patients with depression when used as add-on to medications; however, in spite of obtaining encouraging results, no studies so far investigated its long-term efficacy or the possible role of maintenance dTMS sessions in protecting patients from depressive or manic relapses. We previously reported a 6-month improvement of depression in a patient with rapid cycling BD and protection from mood episodes of any polarity (11).

Given the above considerations, in this preliminary study, we aimed to further assess the efficacy of daily add-on dTMS in relieving drug-resistant bipolar and recurrent unipolar depression, and also to evaluate the role of dTMS maintenance sessions in protecting patients from developing depressive or manic relapses during a 12-month follow-up period.

## Materials and Methods

### Patients

The study was conducted at the Psychiatric Unit of the Sant’Andrea University Hospital, Sapienza University, Rome, Italy, and at the Neuropsychiatric Unit, Villa Rosa Hospital, Viterbo. Recruitment was carried-out from October 2011 to April 2013; 24 consecutive drug-resistant, right-handed patients (13 male, 11 female) with a current DSM-IV-TR depressive episode were enrolled. All patients were Caucasian. Eight had BD Type I (all from Rome), seven had BD Type II (one from Viterbo, others from Rome), and nine had MDD diagnosis (three patients from Viterbo, others from Rome). Patients had no other psychiatric comorbidity. Diagnoses were established through the Structured Clinical Interviews for DSM-IV Axis I (SCID-I) and II (SCID-II); details are given in Table 1. All patients gave written informed consent for participation in the study and subsequent publication of results. The study received the approval of the local ethical boards (human experimentation ethics committee of Sapienza University, Rome, Italy, and Villa Rosa, Viterbo).

**Table 1:** **Participants’ clinical and sociodemographic characteristics**.

	Patients with dTMS maintenance (*N* = 12), mean ± SD	Patients without dTMS maintenance, *N* = 12, mean ± SD	Mann–Whitney *U*-test	χ^2^	*P*
Age	47.70 ± 8.90	48.50 ± 14.26	59.5		0.478
Men, *N* (%)	6 (50)	5 (41.7)			0.682^a^
DSM-IV-TR diagnosis
BD I, *N* (%)	4 (33.3)	4 (33.3)		
BD II, *N* (%)	4 (33.3)	3 (25)		
MDD, *N* (%)	4 (33.3)	5 (41.7)		0.254	0.881
Duration of disease (years)	15.00 ± 9.71	13.10 ± 9.62	60.0		0.514
Drug treatment
Mood stabilizers, *N* (%)	9 (75)	7 (58.3)			0.386^a^
Benzodiazepines, *N* (%)	1 (8.3)	5 (41.7)			0.059^a^
Antidepressants, *N* (%)	5 (41.7)	7 (58.3)			0.414^a^
Two or more psychotropic medications, *N* (%)	3 (25)	4 (33)			0.653^a^
Clinical scales				
Baseline HDRS	23.83 ± 3.27	23.50 ± 3.28	66.5		0.755
Post-dTMS cycle HDRS	9.83 ± 1.27	9.92 ± 2.35	71.5		0.977
HDRS at 6-month follow-up	9.33 ± 2.23	13.75 ± 5.53	34.5		**0.028**
HDRS at 12-month follow-up	12.92 ± 5.78	16.17 ± 7.11	55.5		0.347
Baseline YMRS	2.42 ± 1.16	2.50 ± 0.67	60.0		0.514
Post-dTMS cycle YMRS	2.17 ± 0.58	2.75 ± 0.97	48.0		0.178
YMRS at 6-month follow-up	2.33 ± 0.89	2.83 ± 1.12	56.5		0.378
YMRS at 12-month follow-up	2.66 ± 1.16	3.08 ± 0.52	57.5		0.410

*^a^One-way Fisher exact tests*.

*BD I, Bipolar Disorder Type I; BD II, Bipolar Disorder Type II; MDD, Major depressive disorder; HDRS, Hamilton Depression Rating Scale; YMRS, Young Mania Rating Scale. Significant results in bold characters*.

Inclusion criteria were a current depressive episode and a diagnosis of BD or MDD; age 18–75 years; at least 5-year duration of illness; failure to respond to at least three adequately dosed antidepressants from at least two distinct classes administered for at least 2 months each; availability of reliable informants; wish to participate in the study. Exclusion criteria were concomitant use of substances (with the exception of nicotine, occasional alcohol, and three or less daily cups of coffee or tea); specific contraindications to dTMS (history of seizures, pacemakers, or other electric devices); axis I diagnosis other than MDD and BD; having received dTMS in the past 12 months; left handedness. All patients were on stable drug treatment from at least 1 month. Medication was left unchanged and its details are given in Table 1.

A response of a depressive episode to treatment was defined as an at least 50% drop of Hamilton Depression Rating Scale (HDRS) scores from baseline, a remission was a HDRS score of 7 or less, while a relapse and recurrence were considered the emergence of a fully symptomatic depressive episode after full remission or recovery, respectively (12, 13).

### Clinical measures

Patients were assessed for mood disorder by trained clinicians through the HDRS (14) and through the Young Mania Rating Scale (YMRS) (15) at baseline, at the end of the first cycle of dTMS treatment, after 6 and after 12 months from the end of the first cycle of dTMS treatment.

The HDRS is a 21-item clinician-rated scale designed to assess severity of depressive symptoms; single items are Likert, ranging 0–4 (8 items) or 0–2 (9 items); 0–7 is normal, 8–13 is mild depression, 14–18 moderate depression, 19–22 severe depression, and ≥23 very severe depression; a score of ≤7 indicates remission of symptoms (13). The YMRS is an 11-item clinician-rated scale designed to assess the severity of manic symptoms; 4 of the YMRS items are rated on a 0–8 scale, with the remaining 5 items being rated on a 0–4 scale; a score of ≤12 indicates no mania or remission of symptoms (15).

### Deep TMS protocol

For dTMS sessions, we used Brainsway’s H1 coil deep TMS System (Brainsway, Har Hotzvim, Jerusalem, Israel). The H1 coil is designed to elicit neuronal activation in medial and lateral prefrontal regions, including the orbitofrontal cortex, with a preference for the left hemisphere (16). H1 coils were positioned over patients’ scalp. The optimal spot on the scalp for stimulation of the right *abductor pollicis brevis* muscle was located, and the motor threshold established by delivering single stimulations to the motor cortex. The motor threshold, defined as the lowest stimulation intensity producing five motor evoked potentials (MEPs) of at least 50 μV in 5 of 10 stimulations, was measured by gradually increasing stimulation intensity. The site of stimulation was located 5.5 cm anterior to the point at which maximum stimulation of the *abductor pollicis brevis* muscle was reached. dTMS treatment was delivered by three expert, trained, certified physicians (CR, VRF, SDP) in 20-min sessions. Each patient received 55 18-Hz trains per session at 120% of the measured motor threshold, with 2-s duration each and 20 s inter-train intervals, for a total of 1,980 pulses per session. The complete cycle of the dTMS treatment consisted of 5 consecutive session days in a week for 4 consecutive weeks, for a total of 20 sessions amounting to 39,600 pulses for each patient. These parameters are the same as our research team endorsed in treating clinically depressed patients (17–19) and are recommended by the manufacturers.

The first complete cycle of the dTMS treatment consisted of 5 consecutive session days in a week for 4 consecutive weeks, for a total of 20 sessions amounting to 39,600 pulses for each patient. After the first dTMS cycle, patients were randomly assigned to two groups, one receiving maintenance dTMS sessions (M), and another, which returned to background pharmacotherapy with no further dTMS sessions (non-maintenance group, NM). The M group received 2 sessions per week during the first month after the conclusion of the regular dTMS cycle and 1 weekly session for the subsequent 2 months for a total of 16 sessions after the 20 regular dTMS sessions. Other measures related to the session, i.e., frequency, number of pulses and trains, stimulus duration, and inter-train intervals were as in the regular sessions.

### Statistical analysis

All analyses were performed with SPSS 19.0 statistical package for the social sciences (IBM, Armonk, NY, USA). The Mann–Whitney *U*-test was used for the between-groups comparison (M vs. NM patients); the non-parametric Wilcoxon Sign Test was used for within-groups comparisons (pre-dTMS vs. post-dTMS; post-dTMS vs. 6-month follow-up; post-dTMS vs. 12-month follow-up). The use of the non-parametric tests was chosen because none of the examined variables were normally distributed (Shapiro–Wilk test, *p* < 0.05) in all considered conditions. Finally, one-way Fisher exact tests and Chi-squared tests (χ^2^) for *N* × *N* contingency tables were used.

## Results

Drug treatment and other baseline patient characteristics are given in Table 1. M and NM groups did not significantly differ for gender, age, duration of disease, and drug treatment, as well as for baseline and post-treatment HDRS and YMRS scores (Table 1). Compared to NM patients, M patients scored lower on the HDRS at the 6-month follow-up (13.75 ± 5.53 vs. 9.33 ± 2.23;*p* = 0.028); there were no significant between-groups differences in HDRS mean scores at the 12-month follow-up (Table 1). YMRS scores between M and NM did not differ significantly at both 6- and 12-month follow-up time-points (Table 1).

After the first dTMS treatment sessions, a significant reduction of HDRS scores was observed in both M (pre-dTMS HDRS: 23.83 ± 3.27; post-dTMS HDRS: 9.83 ± 1.27; *p* = 0.002) and NM groups (pre-dTMS HDRS: 23.50 ± 3.28; post-dTMS HDRS: 9.92 ± 2.35; *p* = 0.002) (Table 2). However, while in M, the HDRS mean scores did not significantly change at the 6- and 12-month follow-up (Table 2), a significant increase of HDRS occurred in NM at the 6-month follow-up (post-dTMS HDRS: 9.92 ± 2.35; 6-month HDRS: 13.75 ± 5.53; *p* = 0.046), which were further strengthened at the 12-month follow-up (post-dTMS HDRS: 9.92 ± 2.35; 12-month HDRS: 16.17 ± 7.11; *p* = 0.040) (Table 2).

**Table 2:** **Within-groups HDRS changes after dTMS, at the 6-month follow-up, and at the 12-month follow-up**.

	Patients with dTMS maintenance, mean ± SD	*P*	Patients without dTMS maintenance, mean ± SD	*P*
Baseline HDRS	23.83 ± 3.27		23.50 ± 3.28	
Post-dTMS HDRS	9.83 ± 1.27		9.92 ± 2.35	
Wilcoxon-*Z* Test	−3.070	**0.002**	−3.083	**0.002**
Post-dTMS HDRS	9.83 ± 1.27		9.92 ± 2.35	
HDRS at 6-month follow-up	9.33 ± 2.23		13.75 ± 5.53	
Wilcoxon-*Z* Test	−0.770	0.441	−1.995	**0.046**
Post-dTMS HDRS	9.83 ± 1.27		9.92 ± 2.35	
HDRS at 12-month follow-up	12.92 ± 5.78		16.17 ± 7.11	
Wilcoxon-*Z* Test	−1.146	0.252	−2.050	**0.040**

*Significant results in bold characters*.

## Discussion

Deep transcranial magnetic stimulation is a promising new treatment for resistant depression, but it has still to gain a foothold in current practice. Studies heretofore have shown its efficacy as add-on and its safety, but the general impression is one of a fleeting effect. The idea of maintenance sessions came from the practice of electroconvulsive therapy. The only study in literature that envisaged maintenance was an open-label study and reported positive results, provided that negative cognitive–emotional reactivation is kept low (20). Our study confirmed previous evidence of a possible positive therapeutic effect of H-coil dTMS on depressive symptoms in BD and MDD when added on ongoing treatment with mood stabilizers and antidepressants. The significant decrease of HDRS scores after the first dTMS cycle (Tables 1 and 2) pointed to improvement of depression.

Our results also suggest that, after remission/recovery from an acute episode, maintenance dTMS sessions may be helpful in preventing depressive or manic relapses and maintaining euthymia in a 12-month follow-up period. In fact, while the patients with no continuation dTMS sessions showed a gradual significant increase in HDRS scores after 6 and 12 months, the HDRS scores of patients who underwent maintenance dTMS did not significantly change over time (Table 2). In addition, patients who underwent maintenance dTMS therapy scored significantly lower on the HDRS with respect to patients randomized to receive no additional dTMS sessions after 6 months (Table 1). Scores on the YMRS did not change significantly throughout the study in both M and NM groups; the two groups did not differ on the YMRS at any time-point through the 12-month observation period, indicating the absence of significant manic/hypomanic symptomatology in patients with unipolar or bipolar depression who were exposed to a full, 20-session dTMS cycle.

The results of the present research suggest the possible utility of add-on dTMS in patients with treatment-resistant depression for both inducing antidepressant response and episode remission/recovery, and for obtaining relapse/recurrence prevention. This approach, which integrates medications and dTMS, may greatly impact the treatment of MDD and BD, as both disorders are characterized by high rates of treatment-resistance and relapse (21).

Since this was an add-on study, patients were concurrently receiving constant doses of mood stabilizers and antidepressants throughout the dTMS treatment period. The type of medications varied widely, hence we cannot draw conclusions about the possible influence of any given drug on the antidepressant efficacy elicited by dTMS; however, all these drugs and drug combinations were ineffective at least for the last month before initiating dTMS.

We are not able to speculate with strong arguments about the protective effect of maintenance dTMS against depressive episodes and more so, against manic episodes. There is evidence for other somatic treatments, like electroconvulsive therapy, as inducers of manic switches in unipolar or bipolar patients with depression (22); we may state that this problem did not emerge with dTMS in our population, but since no patient presented significant variations in YMRS scores in either M or NM groups, we may not infer as to the value of maintenance dTMS in protecting against manic episodes, but only remark the low propensity of dTMS in the long run to associate with mood elevations. Regarding a putative maintenance dTMS-mediated protection from depressive mood episodes, we observed a decreased incidence of relapses/recurrences in the M group compared to the NM group, and also a not significant elevation of HRDS scores in the former group, compared to significant elevations at both 6- and 12-month follow-up in the NM group. However, the sample size was low and it might have supported the creation of an artifact; in fact, similar differences from an arbitrarily chosen second baseline (the HDRS score at the completion of the regular cycle) did not yield statistical significance for the M group at the 12-month follow-up (9.83 ± 1.27 vs. 12.92 ± 5.78, *p* = 0.252) while they did for the NM group at the 6-month follow-up (9.92 ± 2.35 vs. 13.75 ± 5.53, *p* = 0.046). We may speculate that maintenance had a role in preventing depressive episodes, but that there is a tendency for this effect to be lost after some time. Studies using maintenance strategies involving more continuous “recall” sessions (for example, once a month or so) could inform us better about the potential of maintenance dTMS to avoid the relapse/recurrence of depressive episodes.

No serious adverse events occurred during the trial. Some patients reported only mild and transient side effects such as nausea, diaphoresis, mild headache, and scalp discomfort. This is consistent with literature on the effects of dTMS on neuropsychiatric disturbances (9, 23–26).

These data extend the antidepressant efficacy of add-on dTMS that we obtained in the past in comorbid alcohol use disorder and dysthymic disorder (18, 19) to major depression and BD. It appears that resting left DLPFC perfusion is key indicator of later antidepressant response to left DLPFC stimulation (27).

### Study limitations

The open design, the small sample size, a possible confounding effect of add-on medication, the lack of a sham control, the population heterogeneity, the two heterogeneous sites involved constitute the major limitations of this preliminary study. Furthermore, we excluded left handers on the grounds that we should change the parameters of our device to stimulate the dominant hemisphere in that population, and this was not currently feasible [or, alternatively, to decrease stimulus frequency below 1 Hz to account for the proposed hemisphere imbalance in depression (5)]. Thus, our results should be considered as preliminary. Nevertheless, they point toward a new frontier in the long-term treatment of depressive symptomatology, regardless of diagnostic category. Future studies should use larger and more homogeneous samples with double-blind designs and address problems related with handedness to better assess the potential efficacy of dTMS in the treatment and the prevention of depressive episodes in mood disorders.

## Conflict of Interest Statement

In the past two years, Paolo Girardi has received research support from Lilly, Janssen, and Springer Healthcare, and has participated in Advisory Boards for Lilly, Otsuka, Pfizer, Schering, and Springer Healthcare and received honoraria from Lilly and Springer Healthcare. Abraham Zangen is consultant for and has financial interests in Brainsway Inc., which has commercial interests in the development of deep TMS coils. Ruggero N. Raccah is scientific consultant to ATID Ltd, distributor of deep r-TMS (Brainsway) technology in Italy. All other authors of this paper have no relevant affiliations or financial involvement with any organisation or entity with a financial interest in, or financial conflict with the subject matter or materials discussed in the manuscript. This includes employment, consultancies, honoraria, stock ownership or options, expert testimony, grants or patents received or pending, or royalties. This work has not been supported by any funding.
